# Virtual engagement of under-resourced communities: Lessons learned during the COVID-19 pandemic for creating crisis-resistant research infrastructure

**DOI:** 10.1017/cts.2022.385

**Published:** 2022-04-04

**Authors:** Andrew D. Plunk, Alexandra Carver, Charles Minggia, Kassandra Prasanna, Brynn E. Sheehan, Matthew Herman, Cynthia B. Burwell, F. Gerard Moeller, Alex H. Krist, Ethlyn McQueen-Gibson

**Affiliations:** 1 Department of Pediatrics, Eastern Virginia Medical School, Norfolk, VA, USA; 2 Office of Diversity and Inclusion, Eastern Virginia Medical School, Norfolk, VA, USA; 3 Department of Psychiatry and Behavioral Sciences, Eastern Virginia Medical School, Norfolk, VA, USA; 4 Healthcare Analytics and Delivery Science Institute, Eastern Virginia Medical School, Norfolk, VA, USA; 5 Department of Health, Physical Education and Exercise Science, Norfolk State University, Norfolk, VA, USA; 6 Wright Center for Clinical and Translational Research, Virginia Commonwealth University, Richmond, VA, USA; 7 School of Medicine, Virginia Commonwealth University, Richmond, VA, USA; 8 Department of Family Medicine and Population Health, Virginia Commonwealth University, Richmond, VA, USA; 9 School of Nursing, Hampton University, Hampton, VA, USA

**Keywords:** Community engagement, digital methods, digital inclusion, community advisory board, mixed-methods research

## Abstract

The COVID-19 pandemic led to an increased need to conduct research and community engagement using digital methods. Unfortunately, the shift away from in-person research activities can make it difficult to engage and recruit participants from under-resourced communities that lack adequate digital infrastructure. At the beginning of the pandemic, our team recognized that imminent lockdowns would significantly disrupt ongoing engagement with low-income housing resident community partners and that we would ultimately bear responsibility if that occurred. This manuscript outlines the development of methods designed to create capacity for virtual engagement with a community advisory board that were subsequently applied to a longitudinal mixed-methods study. We describe how our experience engaging low-income housing residents during the height of the pandemic influenced the approach and offer guidelines useful for engaging under-resourced communities regardless of setting. Of these, a strong commitment to providing technology, unlimited data connectivity, and basic digital literacy training/technical support is most important. While each of these is essential and failure in any one area will reduce overall effectiveness of the effort, providing adequate technical support while maintaining ongoing relationships with community members is the most important and resource-intensive.

## Introduction

The COVID-19 pandemic is a public health crisis that has presented numerous challenges to conducting research, particularly community-engaged research. Notably, the transition to digital methods is problematic due to the potential to disrupt participation in research that has traditionally relied on face-to-face contact [1].

Unfortunately, the shift from in-person to online video communication during the COVID-19 pandemic has been especially burdensome for individuals from under-resourced, lower-income communities. Digital access and skills are important social determinants of health [2] operating at multiple levels, such as individual (e.g., digital literacy and device ownership), family (e.g., a private and secure space), and community (e.g., digital infrastructure and access) [3]. While these factors create barriers for all forms of digital inclusion, including telehealth, distance learning, and telework, there are additional challenges for virtual research since participation is not a considered a priority for many communities. We sought to overcome these barriers through engagement and relationship building.

## Development of a Virtual Community Engagement Protocol

### Pre-Pandemic Community-Engaged Research Infrastructure

For nearly a decade, investigators from Eastern Virginia Medical School (EVMS) have met regularly with a community advisory board (CAB), comprised of Norfolk, VA, low-income housing residents. The CAB has been involved in several grant-funded projects, initially focused on respiratory health, particularly childhood asthma, and later on studies examining smoke-free public housing [e.g., 4–6]. CAB members are active partners contributing to all stages of research, including dissemination efforts. Prior to the pandemic, there were approximately 15 CAB members in regular attendance at monthly in-person meetings on the EVMS campus with transportation provided by the local public housing authority.

### Recognizing a Responsibility to Maintain Contact with Under-Resourced Community Partners

There was a rapid shift from in-person to virtual research methods and telehealth at the start of the COVID-19 pandemic. However, our team recognized that the realities of the digital divide meant that naïvely transitioning to online meetings with our CAB partners was untenable (e.g., several Norfolk CAB members had no prior experience with smartphones or computers). Absent immediate and drastic action, the pandemic would significantly disrupt our ability to engage with community partners. Simultaneously, these individuals also represented a population extremely vulnerable to COVID-19. We were very concerned that communities most in need of engagement during the health crisis would be systemically excluded from the pandemic response, with efforts biased toward healthier populations with preexisting technical expertise (e.g., younger and with higher educational attainment). It was in this context that we began to embrace the obligation for creating the capacity for our community partners to interact with us.

### Initial Virtual Interactions and Lessons Learned

Recognizing the inevitable shift to virtual communication, low-cost laptop computers were ordered for CAB members on March 14, 2020, 2 days before in-person engagement was prohibited. Laptops were handed out to all CAB members by March 27, 2020 with the first virtual meeting held on March 31 using the teleconferencing platform Zoom. Unfortunately, the first few meetings were unsuccessful as the laptops were “too much” for CAB members unfamiliar with technology, who expressed the need for a simpler interface. Further, the cameras were of relatively poor quality. CAB members with smartphones had access to much better cameras, but this raised concerns about the use of members’ cellular data, almost all of whom had limited data plans. There were several instances of CAB members using all their data for a video call early in the month and not having internet access until their next billing cycle.

#### Broadband and internet access

While the team had anticipated issues with technology, we had not fully appreciated how limiting a lack of access to broadband would be. In the end, only a handful of CAB members who already had cable internet were able to use their laptops at all and, of these, most transitioned to using smartphones after learning how to connect them to existing Wi-Fi. The remaining CAB members dialed into Zoom meetings, essentially creating a conference call. However, this introduced other barriers. CAB members were unfamiliar with teleconferencing etiquette and those without the ability to use visual cues from video were at an additional disadvantage and consistently spoke over each other. Further, those without the ability (or technical know-how) to mute their phones were more disruptive to the group conversation (notably, these issues resolved themselves once all CAB members were able to use Zoom). Our temporary solution, which lasted for several months, was to have multiple meetings with subgroups of the CAB, one for members with broadband, and a second for those who had to dial in telephonically. However, this only reinforced different experiences for those with less technical ability and fewer resources – in essence doing that which we were trying to avoid.

It was obvious that all CAB members needed unlimited internet access and a device with a high-quality camera. The team explored cable internet as an option; however, it was difficult to consistently subsidize the cost of cable internet while keeping the account in the CAB member’s name. Prior unpaid cable television/internet bills were relatively common and often precluded reactivating the account without first paying the balance. Although paying for ongoing costs of CAB members’ internet would be justified and permitted, our institution would not allow us to pay past-due cable bills.

Cellular service, which we began experimenting with in September 2020, was a more appealing option. The process was streamlined with integrated data/device plans making billing and account management simpler. In October 2020, we procured tablet computers with unlimited data from a cellular carrier, who offered substantial discounts as part of a program designed for virtual learning. However, we discovered it is best not to rely on a single service provider, as some sites will not have good coverage with the first choice of carrier. Cellular hotspots with a second carrier were obtained to address service gaps. This combination – tablets with unlimited data and cellular hotspots as a backup – is currently our preferred approach and has provided usable virtual access to the majority of our CAB members.

#### Digital literacy

Following initial virtual interactions with the CAB, it became clear that members would benefit from both technical support and digital literacy training. Even those who we expected to have greater familiarity with technology experienced problems when faced with unfamiliar platforms or an unreliable connection. A proactive approach was needed to ensure that lack of digital literacy did not become an additional barrier to engagement. Further, we realized this effort had to be grounded in our relationships with the CAB so that they were truly comfortable receiving ongoing support. For example, we began calling CAB members with recurrent issues 30 minutes before each Zoom meeting to guide them through the process of connecting. We also developed a system in which several staff were available at the beginning of each meeting for technical support via phone for anyone experiencing difficulty using the platform. By early 2021, we had begun creating standardized technical support procedures, which included a primary staff point of contact for technical support to coordinate and document these efforts. By mid-2021, processes outlining solutions to common problems were readily accessible to team members.

### CAB Expansion

#### CAB member recruitment

Before the pandemic, our CAB recruitment process began with sharing project goals and intentions with community members. Restrictions on face-to-face contact required that we begin the process by asking housing authority staff and existing CAB members for recommendations. We also recruited residents using flyers placed in mailboxes and posted in high traffic areas of apartment buildings. Interested individuals were called by a research staff member, who provided information about the CAB’s goals, topics of discussion, CAB member responsibilities, and incentives for participating. All CAB members received the free tablet computer with internet and $10 per hour for every meeting attended.

#### Tablet preparation

We discovered that setting up tablets before delivery addressed many technical support issues before they became problematic. Initial preparation included inserting SIM cards, charging the battery, and turning on the tablet. A Google account with an email address was also created for each CAB member. Over time, we developed a formal protocol with the following additional steps: (1) downloading relevant apps and creating accounts for them, (2) enabling accessibility options that we have identified should be changed by default (e.g., increasing screen timeout delay), (3) assessing whether participants need other accessibility options enabled, such as enlarged fonts, magnifier window, and screen zoom, (4) moving the icons for the Zoom and Messages apps to the home screen for easy access, and (5) updating several Zoom options (e.g., enabling “Auto-Connect to Audio” to streamline connecting to meetings). Each tablet was then cleaned and inserted back into its original box, which was placed in a bag with a tablet case, a “refrigerator flyer,” a W-9 form (or a W-9 waiver if payment is declined), and a copy of a tablet agreement form, which stipulates how the tablet can be used. The refrigerator flyer contains the PIN to unlock the tablet, email address created for the participant, passwords for both Google and Zoom accounts, and Google Voice phone numbers of research team staff.

#### Onboarding

Research staff scheduled one in-person meeting with interested residents, which was always held outside to minimize potential exposure to COVID-19, to complete required paperwork and drop off the tablet. A follow-up phone/virtual meeting was then scheduled to walk new CAB members through their first Zoom meeting.

### Application of the Approach to a Longitudinal Mixed-Methods Study of Mistrust in COVID-19 Guidance

We discovered that methods created to sustain virtual engagement with under-resourced community CABs could be used for other types of research activities, which was appealing due to ongoing limitations on face-to-face contact with participants. Due to the resources needed to create capacity and provide digital literacy training, we opted for a longitudinal design with repeated interaction with the same cohort of participants over time. With this infrastructure, we have been conducting mixed-methods research on how mistrust affects compliance with COVID-19 guidance that combines qualitative methods using focus groups or individual interviews on platforms like Zoom with the ability to collect quantitative data using the same device (e.g., by sending links to online assessments).

### Cohort recruitment, tablet preparation, delivery, and onboarding

These initial steps were modeled after the process developed for our CAB members, with the primary difference being that written informed consent was obtained during tablet delivery.

#### Initial demographic interview

After tablet delivery, staff administered an initial assessment to participants using Zoom in a one-on-one interview format to give them practice logging in and gain familiarity with the platform before asking them to participate in a group activity.

#### Focus group discussions

Our current focus group protocol contains three phases: (1) focus group scheduling and preparation, (2) conducting the virtual focus group discussion, and (3) post focus group discussion.

##### Focus group scheduling and preparation

In order to maintain participant availability, after recruiting a new participant, staff ask them about their ongoing availability for a 1–1.5 hour-long focus group discussion. Availability is saved for each participant and updated on an as-needed basis (typically when a participant indicates they are no longer available during that time). Participants are then asked to complete up to four study activities per month, at least one of which should be a focus group discussion (the cohort is sampled separately for each topic). Already-completed activities and participant availability determine scheduling, with new focus group topics made available on a rolling basis. After identifying 6–9 participants with similar availability, a date and time are selected for the focus group discussion. Focus groups are scheduled during the day, evenings, and on weekends to ensure all participants have opportunities to contribute. Selected participants are called and invited to attend. Participants that agree to join are sent Zoom meeting details to their phones and tablets. A reminder is sent the day of the meeting and participants who have had prior technical support issues are called 30 minutes before the meeting to ensure that they are able to connect. Connection tests are conducted with participants on an as-needed basis. Staff are required to test their connection prior to the scheduled meeting if joining from a new location or computer.

##### Conducting the virtual focus group discussion

Each focus group includes a designated facilitator who directs the discussion, while two other staff coordinate with participants, introduce the session, obtain participant consent, take notes, obtain audio recordings, and provide technical support during the meeting. All staff join the Zoom meeting room 5 minutes early to identify participants as they log on. The meeting host begins recording once participants have joined the meeting room. A redundant audio recording is obtained using a handheld recorder to capture the conversation through their computer’s speakers. Throughout the duration of the focus group, research staff attempt to identify, contact, and assist any individuals who may still need technical assistance. Participants are also provided a phone number to reach one of the research staff for technical support. Research staff first greet and lead introductions of the team and participants before reading a consent script and reviewing Zoom etiquette guidelines (e.g., reminding participants to be aware of their surroundings, as other attendees will be able to see and hear them). The facilitator will then begin following the discussion guide.

##### Post focus group discussion

At the conclusion of the focus group, the team meets to discuss the session. Field notes are also completed at this time. Field notes, video, and audio files are saved to the team’s internal server. The audio file is reviewed and submitted for professional transcription. Once transcripts are available, they are edited to remove identifiable information. Participant names are changed to their study ID. Minor formatting is also done at this time (e.g., bolding questions asked by the facilitator). Additionally, participant activities are added to a tracking form that is processed once per month for participant payment.

#### Survey assessment

Participants are asked to complete 2–4 survey assessments each month, which cover a range of topics related to project goals. Assessments are administered through REDCap using the platform Twilio, which allows survey links to be texted to the Messenger app on participants’ tablets. Links are unique to each participant and allow their responses to be easily tracked across the project.

### Success of the Virtual CAB

We have met with the CAB at least once per week since the pandemic began. By December 2021, we had held over 180 virtual meetings with CAB members to whom we provided technology. Attendance has improved relative to pre-pandemic meetings. CAB members cited convenience (e.g., not having to travel), reduced time commitment per meeting, and the value of the meetings as a social outlet during the pandemic.

The shift to an online platform also allowed us to expand the reach of the CAB, despite limitations on face-to-face contact. We first increased our regional presence to include the Virginia cities of Chesapeake, Hampton, Portsmouth, Newport News, Suffolk, and Virginia Beach, in addition to Norfolk. This process took 3 months. We have also since expanded into other regions in Virginia, with additional members from the cities of Richmond and Roanoke. There are currently 28 CAB members, all of whom receive a housing benefit from an agency in one of these cities. Notably, these jurisdictions represent both urban and rural areas and, taken together, administer 49% of federally assisted low-income housing in the state [7].

### Success of the Virtual Longitudinal Mixed-Methods Study of Mistrust in COVID-19 Guidance

Since beginning the study, 205 individuals were either referred or contacted us directly with 135 becoming fully enrolled. Inclusion criteria included being at least 18 years old and a resident of one of the partnering public housing communities. Primary reasons for declining participation after contact were competing obligations or lack of desire to participate in activities on a recurring basis. Participant ages ranged from 18 to 75 years of age, with a mean age of 53 years. Many of our participants share similar racial backgrounds, with 92% (n = 110) being African American. A smaller number of participants identified as White (n = 5), Biracial or Multiracial (n = 2), or of Hispanic, Latinx, or Spanish origin (n = 6).

Participant retention has relied heavily on regular engagement and relationship building strategies. To date, we have retained 87% of enrolled participants, a rate that compares favorably to the 74% average for published longitudinal studies [8]. When a participant does withdraw, they have on average been replaced within a month. Over time, this has allowed us to maintain a cohort of participants at or near our sample size goals (i.e., 95% or higher). Of those who did leave the study, all but five did so before completing all of the initial onboarding process, emphasizing the importance of relationship building and gaining early buy-in. While participants who withdraw from the study keep their tablets, their internet service is transferred to another device, which is then offered to a new participant.

This approach has allowed us to complete over 1,200 total data collection activities with the cohort, including one-on-one interviews, focus group discussions, and online surveys. Expanding these methods to the longitudinal study led to numerous refinements, particularly with providing technical support, that have in turn benefited engagement with the CAB.

## Discussion

Based on our experience creating the infrastructure to support virtual community engagement and mixed-methods research, we provide recommendations for virtual engagement of under-resourced communities in Table 1. A commitment to providing technology, data connectivity, and technical support is needed when community capacity in these areas is limited. Of these, technical support is the most time- and resource-intensive, as this should occur in the context of maintaining ongoing relationships with community members. Unfortunately, our experience is that researchers do not adequately plan for this need. It is important to understand that ongoing technical support will be required with any virtual engagement, even when it is assumed that community members are technologically proficient.

**Table 1.** Recommendations for virtually engaging under-resourced communities

Commit to providing technology, data connectivity, and technical support as part of ongoing relationships with community members.	
* Provide a tablet with HD webcam as part of an integrated cellular service/device plan*	Use the same model of tablet, if possible – having a standard platform will make technical support easier.
* Have a backup option for internet service*	While tablets with cellular service will provide connectivity for most participants, investigators should expect localized problems that will be very disruptive for some individuals. A wireless hotspot with a different provider is a good option and investigators should expect to help participants connect their tablet to the hotspot.
* Provide technical support and digital literacy training*	Unresolved technical issues are very disruptive, both to activities while they are occurring (e.g., during a focus group meeting) and to engagement over time, as participants will quickly become frustrated. Most technical support can be done over the phone, and our goal is to resolve issues without having to resort to an in-person visit. Common problems can also be addressed during tablet drop off by showing participants basic functionality in person (e.g., “swiping”).
* Develop standardized support processes*	Identify staff point of contact for standardizing technical support. New issues should be escalated to the designated staff member rather than taking other staff time. Emergent issues are documented so that other staff can provide support in the future.
Use a common videoconferencing platform	We recommend Zoom, as it has become fairly standard and has robust features. Breakout rooms are especially important, as they offer a way to discuss divisive topics in a smaller group setting. Regardless of the platform chosen, avoid changing how participants interact with the team, as this will cause confusion.
Check-in with participants regularly (at least weekly)	Communicating with participants on an ongoing basis is pivotal to relationship building and maintaining participation across multiple months. While the tablets themselves offer additional modes of communication, in practice most of this is done via phone by calling or texting, as many participants are less comfortable using a tablet and do not regularly check email.
Moderate discussions with privacy as a priority	Remind participants to keep group discussions private, preferably by being alone or using headphones so others in their space cannot hear the conversation. Participants will often forget during the course of the meeting that they can be heard and seen by the whole group. Active moderation is required when a participant begins to have a private conversation with someone (e.g., a visitor at their door) or brings their tablet into a private space with the camera on (e.g., a bathroom).
Reduce pressure to provide “correct” answers	Likely due to the ease of accessing information, participants have on several occasions found answers online when responding to knowledge-based questions. Focusing on opinion-based questions seems to decrease perceived pressure to be “right” and produces more authentic responses.

While virtual engagement and research activities have been of fundamental importance in response to COVID-19, we anticipate that these methods will remain useful after the pandemic ends. Face-to-face engagement will likely be preferred by many researchers when it can be done safely due to concerns about creating bias based on digital inclusion, but the convenience of virtual methods coupled with the potential for engaging broader geographic areas is appealing. We have been able to engage many more communities than would have been possible had we remained reliant on in-person engagement. This increases potential for direct positive public health impact while also improving the quality of our research. For example, while generalizability can be a weakness of community-engaged research, it is unlikely that our findings will be limited by the idiosyncrasies of any one specific jurisdiction. Marginalized or under-resourced communities could also be of insufficient size for recruitment of sample sizes required for many research designs, especially outside dense urban centers of major metropolitan areas, and this is by definition true of under-resourced rural communities. In our case, the communities that we have been able to engage represent almost 100,000 of the most vulnerable individuals in our state. This would not have been possible to achieve without the shift to virtual methods.

Crisis-resistant research infrastructure is another area in which virtual methods excel. It would have been difficult to maintain regular contact with our under-resourced community partners during the pandemic without having committed to maintaining relationships and building capacity for virtual engagement. Weekly meetings with the CAB, in particular, have been important for receiving almost real-time feedback regarding CVOID-19 and the US pandemic response.

### Limitations

Our virtual approach is resource- and time-intensive, of which costs associated with providing devices and internet connectivity are the most obvious. Staffing requirements are also relatively high. Both research participants and CAB members require a considerable amount of support initially and while more time will be spent with less technologically savvy individuals, everyone will benefit from a standardized onboarding procedure, which for us currently consists of about a workday per person from start to finish. However, once full onboarding is complete and participants are comfortable navigating the technology, coordinating a 30-member virtual CAB can easily be done by a single staff member devoting approximately 25% of their effort, as the work shifts to maintaining relationships with members and coordinating the actual meetings. Coordinating research participant involvement is more resource-intensive and, once a certain threshold of participants is met, will likely benefit from staff having specialized roles. For example, while we found that one research assistant can coordinate and schedule once-a-week research activities for approximately 50 participants and assist them with basic technical support, it is helpful to have additional team members available to fill roles associated with data collection and management, such as someone devoted to facilitating focus group discussions (of which there will be several per week) and another staff member coordinating the process of texting survey links to participants in REDCap. Our data manager also serves as the point of contact for responding to new technical support issues for which the team has yet to develop a protocol for addressing.

## Conclusion

Community-engaged approaches to capacity building, grounded in authentic relationships, can bridge the digital divide with respect to creating enhanced opportunities for research participation in communities that have in many other ways been left behind by society’s response to the COVID-19 pandemic. Our methods, which reflect a commitment to foundational principles of relationship-focused community engagement in the context of a fundamental shift in how we communicate, will likely remain relevant after pandemic restrictions have been lifted. We anticipate this approach can be exported to other settings to increase recruitment and retention of hard to reach populations.
